# Subsoil microbial community responses to air exposure and legume growth depend on soil properties across different depths

**DOI:** 10.1038/s41598-019-55089-8

**Published:** 2019-12-06

**Authors:** Hongmei Yan, Fan Yang, Jiamin Gao, Ziheng Peng, Weimin Chen

**Affiliations:** State Key Laboratory of Crop Stress Biology in Arid Areas, College of Life Sciences, Northwest A&F University, Yangling, Shaanxi 712100 P.R. China

**Keywords:** Microbial ecology, Microbial ecology

## Abstract

Anthropogenic disturbance, such as agricultural and architectural activities, can greatly influence belowground soil microbes, and thus soil formation and nutrient cycling. The objective of this study was to investigate microbial community variation in deep soils affected by strong disturbances. In present study, twelve soil samples were collected from different depths (0–300 cm) and placed onto the surface. We investigated the structure variation of the microbial community down through the soil profiles in response to disturbance originated by legume plants (robinia and clover) cultivation vs. plant-free controls. The high-throughput sequencing of 16S rRNA genes showed that microbial α-diversity decreased with depth, and that growing both plants significantly impacted the diversity in the topsoil. The soil profile was clustered into three layers: I (0–40 cm), II (40–120 cm), and III (120–300 cm); with significantly different taxa found among them. Soil properties explained a large amount of the variation (23.5%) in the microbial community, and distinct factors affected microbial assembly in the different layers, e.g., available potassium in layer I, pH and total nitrogen in layer II, pH and organic matter in layer III. The prediction of metabolic functions and oxygen requirements indicated that the number of aerobic bacteria increased with more air exposure, which may further accelerate the transformation of nitrogen, sulfur, carbon, and pesticides in the soil. The diversity of soil microorganisms followed a depth-decay pattern, but became higher following legume growth and air exposure, with notable abundance variation of several important bacterial species, mainly belonging to Nitrospira, Verrucomicrobia, and Planctomycetes, and soil properties occurring across the soil profiles.

## Introduction

Microorganisms, a vital soil component, play key roles in the nutrient cycling, organic matter transformation, soil formation^1^, and crop production^2,3^. Some microorganisms clean contaminated soil of organic and inorganic pollutants^4–6^. Soil commonly refers to the 1-m-thick vertical layer below the ground surface^7^, which is developed (formed) through long natural complex processes, and which may be influenced strongly by human activity, environmental change, and soil organic matter^8^. Belowground soil (i.e., 25–200 cm) contains nearly 35% of the total quantity of microbial biomass^9,10^. Recent studies revealed that microbial community composition is strongly affected by soil depth^11^, characterized by highly vertical distributed patterns across the different soil layers^12–15^. Some studies also show that soil physical and chemical properties such as soil carbon, pH, and mineral nutrients, could shape the microbial community occurring in the subsoil^16,17^. The vertical distribution of soil nutrients is dominated by soil texture (clay, silt and sand), anthropogenic disturbances, and weathering dissolution^18^. There is often a pronounced distribution in variation of soil microbial composition across the sampled soil profiles^9,19^, likely because nutrient factors are positively correlated with bacterial diversity^10,20^. It follows that the cycling of soil nitrogen and carbon^21^, as well as moisture content^22,23^, may have profound implications for the stability of microbial communities and the spatial distribution its members. Li, *et al*.^22^ showed that soil pH was higher in the surface layer than in the subsoil of mine tailings profiles; this property was the main factor influencing the bacterial community in that study.

In a soil ecosystem, many biotic or abiotic factors could significantly impact the soil microbial community, such as drought^12^, plant species^19,24^, fertilization and irrigation, pH and soil particle composition^25,26^, and land use and management^27,28^. These factors, many of which are interacting, can lead to a changed ecosystem function through changes in soil microbial community structure and composition^29^. Microbial communities can respond rapidly to environmental changes^18,30,31^. For example, moisture content and the presence of vegetation may enhance microbial community resilience^23,32^. Furthermore, irrigation and fertilization are thought to great affect soil microbial diversity^22,26^. Due to construction activities, deep tillage, and water and soil erosion^33,34^, the surface soil will be stripped and the subsoil exposed to air, which could cause nutrient losses, and thus soil degradation in agricultural fields, now recognized as a global problem. Although the relationship between the diversity of soil microorganisms and nutrient content in arable layer has been widely investigated^11,21,35^, far less known is the response and dynamic patterns of microbial community through the soil profiles to the environmental disturbances in agricultural ecosystem.

To restore soil fertility, chemical or organic amendments are frequently applied^36^. One such preferred measure is growing legume plants to improve the soil nitrogen and organic matter content at a low cost and with a limited impact on the environment. For example, *Korshinsk peashrub* and *Medicago sativa* markedly increased the stock of soil organic carbon and total nitrogen on the China’s Loess Plateau, a place susceptible to wind and water erosion^37^. Faba bean and soybean, when included in rotation with cotton in Australia^38^ or with canola crops in western Canada^39^, improved soil quality by effectively enhancing nitrogen uptake. It is of fundamental importance to explore the dynamics of agro-soil microbial communities in response to plant growth if we are to understand complex microbe-soil-plant interactions in agro-ecosystems.

In the present study, we aimed to investigate the response patterns of agro-soil microbial community through profiles (i.e., 0–300 cm depths) to strong disturbances originated by legume plants (robinia and clover) cultivation vs. plant-free controls. Specifically, we identified the important differential microbial taxa in the surface and subsoil, potentially participating the cycling of nitrogen and carbon, to better understand the microbial process involving in the nutrient cycling in deep soils. Our results determine changes in the deeper soil microbial community in agro-ecosystems in response to environmental disturbances could provide valuable information for restoration of ecosystems and environmental management.

## Materials and Methods

### Sample collection

**Figure 1:**
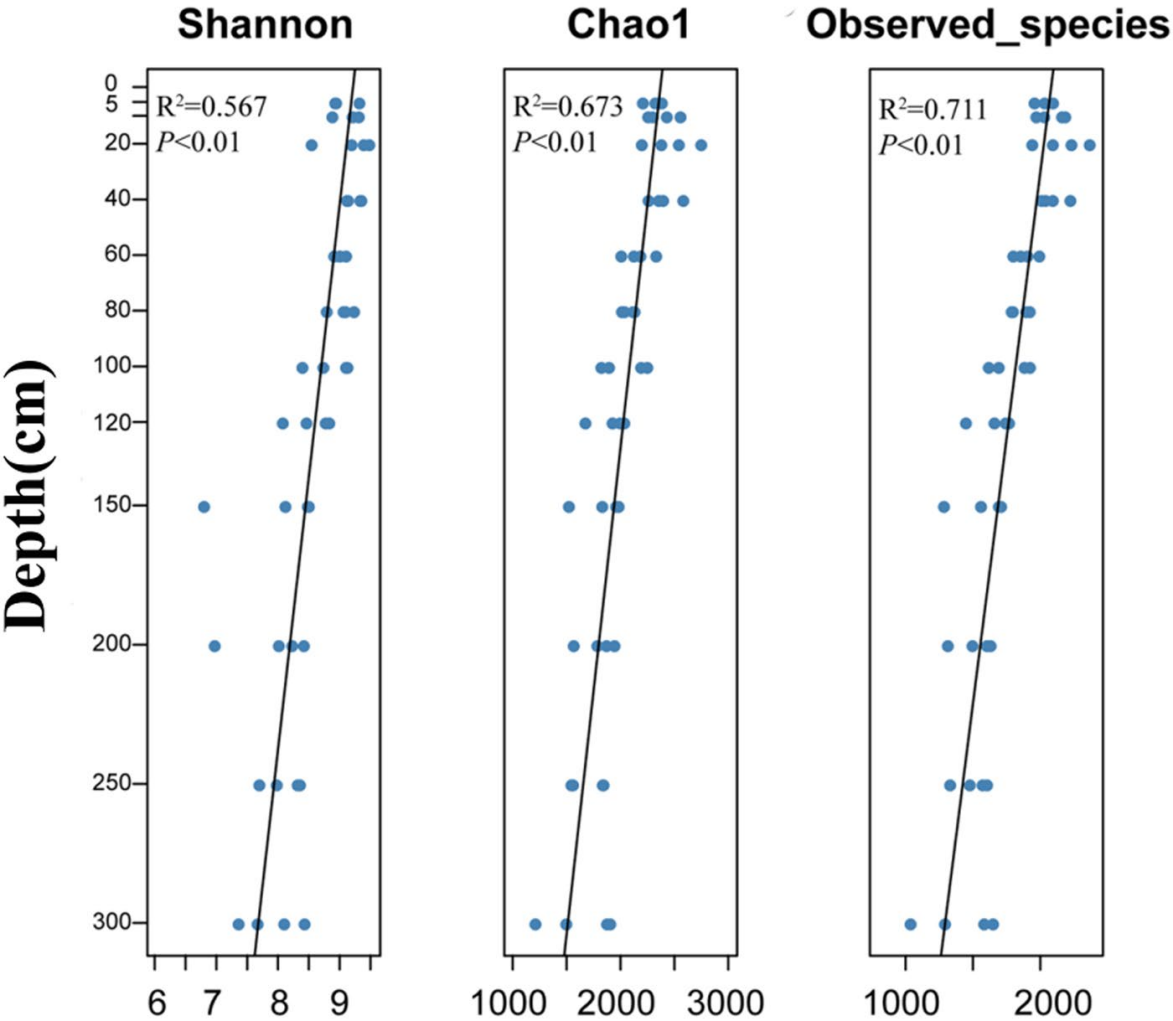
Microbial α-diversity measurements for each depth.

Soil samples were collected with shovel from an agricultural field, a wheat and maize rotation system in Yangling (E108°05′19″, N34°28′84″), on the margin of the Loess Plateau, China. We selected three sites in a 100 × 100 m plot. At each site, a total of 12 soil sub-samples were collected from a 3-m vertical profile, corresponding to depths (cm) of 0–5, 5–10, 10–20, 20–40, 40–60, 60–80, 80–100, 100–120, 120–150, 150–200, 200–250, and 250–300 (Fig. 1). Each soil sub-sample from the three sites taken from the same depth was mixed together, yielding 12 soil samples. From these, a subset of each was stored at –80 °C before further DNA extraction (hereafter, ‘*in situ*’ soil). The remaining soil sample material was taken back to lab in the sealed plastic bags for use in the pot experiments.

### Pot experiments

Pot experiments were performed in greenhouse at the Northwest A&F University (Yangling, Shaanxi Province, China). Three treatments were applied to each soil sample collected: the control (watered only, without plants); growing of *Robinia pseudoacacia* (Robinia); or growing of *Trifolium repens* (Clover). Each fresh soil sample was sieved through 2 mm mesh, and its equivalent 600-g dry weight was put into a 14.5 × 8.5 × 10 cm plastic pot, with sterile water added to reach an 800-g weight per pot. Robinia and clover respectively represent the woody and herbaceous legumes widely grown on the Loess Plateau for ecosystem rehabilitation. All the treatments were replicated three times. We considered all treatments as environmental disturbances, because all the experimental soil was removed from different depths and placed onto the surface, together with the planting and watering.

The seeds of robinia were surface-sterilized by immersing them in a concentrated sulfuric acid (H_2_SO_4_) for 10 min, followed by 95% (v/v) ethanol for 1–2 min, then 10% (v/v) sodium hypochlorite (NaClO) for 10 min. The clover seeds were immersed in 95% (v/v) ethanol for 3 min, and then 50% (v/v) NaClO for 10 min. All seeds of both species were washed at least five times with sterile water. The sterilized seeds were placed on a water-agar medium, and germinated at 28 °C for 36 h. When the seedlings were 1 cm in length, seven robinia or 12 clover seedlings were transplanted per pot, grown in the greenhouse and watered once every 2 days. After 90 days, the plants were harvested and their root length, stem height, nodule number, and dry weight were measured, as were the physical and chemical properties of the potted soil. A subset of soil was collected from each treatment and stored at –80 °C before DNA extraction and the microbial community analysis.

### Soil physical and chemical analysis

Physical and chemical properties of all the soil samples (n = 48)—pH, soil organic matter content (SOM), total nitrogen (TN), available nitrogen (AN), available potassium (AK), and available phosphorus (AP)—were quantified as previously reported^40,41^. Soil texture, including the percent composition of clay, silt, and sand, was determined by a laser diffraction analysis^42^, which was shown in Appendices Table A1.

### 16S rRNA gene sequencing

Genomic DNA was extracted from 48 soil samples (12 soil depths × 4 groups: *in-situ* + Robinia + Clover + Control), by using the MP FastDNA^®^ SPIN Kit for soil (MP Biochemicals, Solon, OH, USA). The V3–V4 hypervariable region of the 16S rRNA gene was amplified in triplicate, by using the primers 341F (5′- CCT AYG GGR BGC ASC AG -3′) and 806R (5′- GGA CTA CNN GGG TAT CTA AT -3′). The purified amplifications from each sample were sequenced on the Illumina MiSeq platform (Illumina Inc., San Diego, CA, USA). The sequences were quality-filtered and chimera-checked by using the Quantitative Insights Into Microbial Ecology (QIIME) workflow. The reads from each of the DNA samples were merged in FLASH software.

### Bioinformatics and statistical analyses

To assess the microbial diversity and abundance, we relied on operational taxonomic units (OTUs) for our analyses, namely of α-diversity and β-diversity, as performed by using QIIME. To estimate α-diversity, the Chao1 richness, Shannon index, and observed number of species per depth of the soil samples were estimated. The β-diversity analysis was done to identify possible correlations between the treatments and the microbial patterns. The weighted Unifrac distance based on phylogenetic information was used to compare the community diversity among samples. A principal coordinate analysis (PCoA), based on the distance matrix, was done to visualize the sample relationships. A constrained analysis of principal coordinates (CAP) was used to reveal the relationships between the microbial taxa and soil properties, and evaluated by a permutation test. We measured the explanatory power of the different explanatory variables in relation to the species structure in the different soil depth layers using a variation partitioning analysis. Differential OTUs were analyzed by fitting a generalized linear model with a negative binomial dispersion method.

The linear discriminant analysis (LDA) effect size (LEfSe) tool was used quantitatively analyze the biomarkers within the different treatments (http://huttenhower.sph.harvard.edu/galaxy/). We performed non-parametric, factorial Kruskal-Wallis (KW) sum-rank test (α = 0.05) to identify those taxa with significant differential abundances between the treatments (using the one-against-all comparisons parameter). Then, the LDA was used to assess the effect size of each biomarker on the treatments divided. We also used the METAGENassist tool database^43^ to predict the metabolic functions and oxygen requirements of the identified genera, as described elsewhere. The functional predictions made in this work are therefore considered only as an indication of the potential microbial functions. We are aware that more throughput tools like shotgun metagenomic analysis yield more robust results. Spearman rank correlations were generated for all the physico-chemical variables and species taxa, with a *P* value cut-off of <0.05.

## Results

### Microbial diversity in the different soil depths

From the *in-situ* soil and the soils disturbed by the Robinia, Clover, and Control (water) treatments, a total of 48 samples were collected from the 0–300-cm vertical profiles for the 16S rRNA gene high-throughput sequencing. On average, they yielded approximately 51,493 effective tags with a read length of 375 bps. The reads were clustered into 4826 OTUs at >97% sequence similarity.

**Figure 2:**
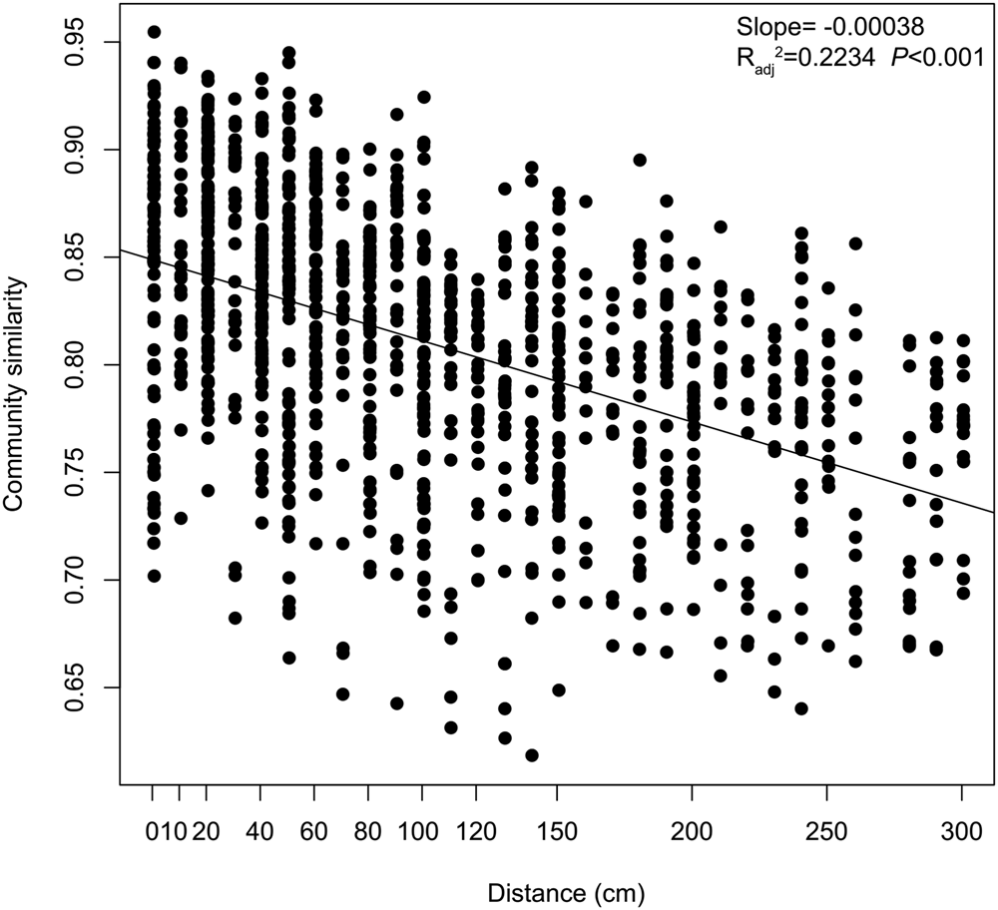
Soil microbial community similarities showed a distance-decay trend, based on weighted UniFrac distance. Regression line: y = –0.00038x + 0.8489.

The linear regressions (Fig. 1) for the Shannon index (*P* < 0.01), Chao1 richness (*P* < 0.01) and Observed species (*P* < 0.01), suggested that microbial α-diversity significantly decreased with soil depth. Furthermore, we investigated the relationship between microbial community similarity and soil depth based on the weighted UniFrac distances. There was a highly distance-decay (or depth-decay) pattern (Fig. 2). We revealed that community diversity had a hierarchical distribution that depended on the soil depth, with a major difference distinguished from layer to I to III (0–40 cm as layer I, 40–120 cm as layer II, 120–300 cm as layer III). In addition, the microbial relative abundances and layers of occupancy were positively correlated, and 30.6% of the OTUs occupied >50% of the depths (Appendices Fig. A1).

### Variation in microbial communities down the three soil layers

**Figure 3:**
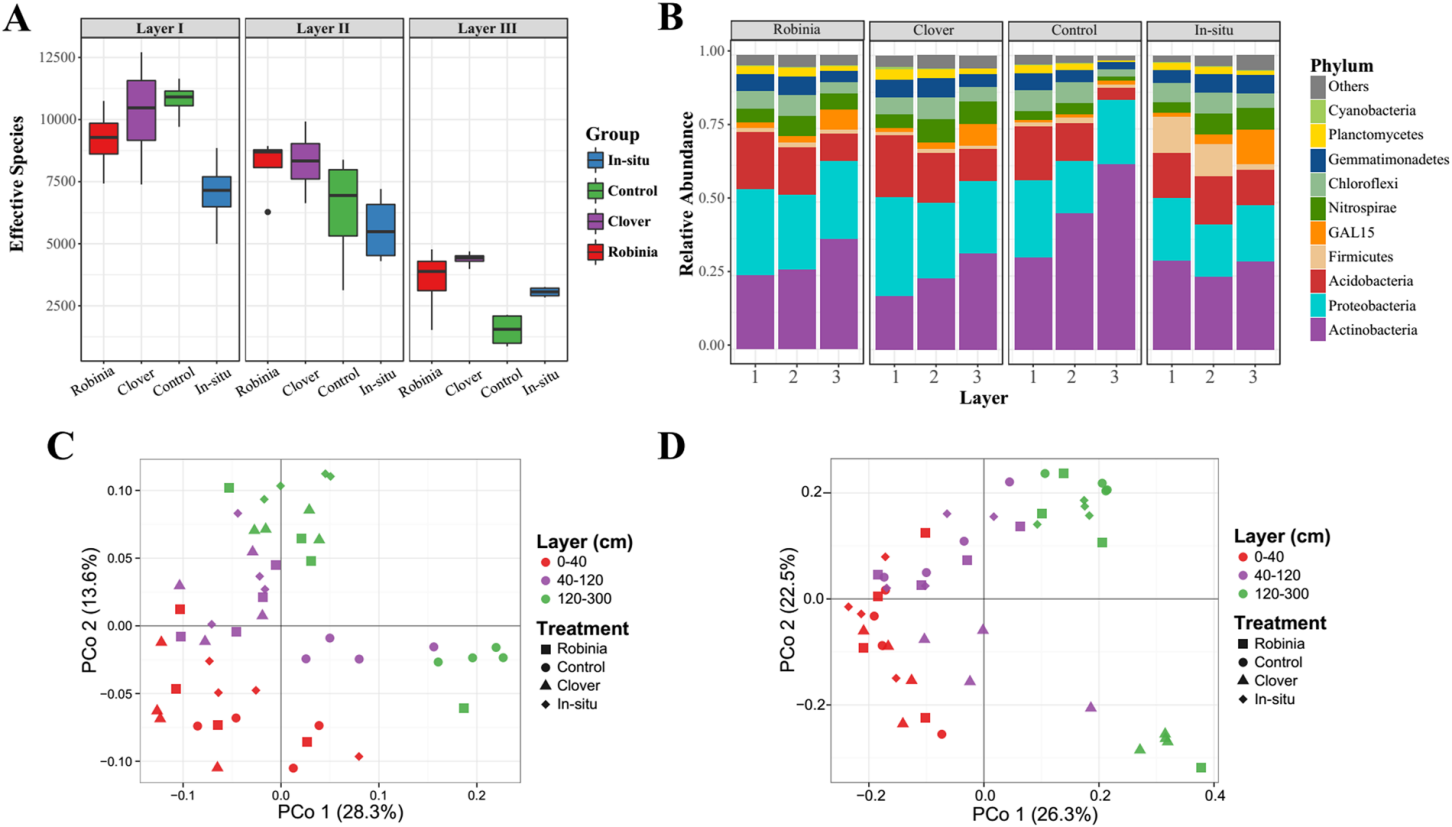
Variation in microbial diversity in the three layers of disturbed soils and *in-situ* soil. (**A**) α-diversity (Shannon) of the three layers sampled from all groups. Estimated species richness was calculated as e^Shannon index^. (**B**) Histograms of the phyla abundances for each treatment group by soil layer. PCoA based on the (**C**) Weighted UniFrac metrics and (**D**) Bray-Curtis metrics.

Microbial α-diversity revealed a gradually lowered diversity from layer I to layer III, and from the disturbed soils to the *in-situ* soil (Fig. 3A). At the phylum level, Actinobacteria, Proteobacteria, and Acidobacteria were dominant populations in each layer for all the soil samples, accounting for >70% in total, and their variation down through the soil profiles was similar between the disturbed and *in-situ* soils. For example, Actinobacteria increased significantly with depth, whereas Proteobacteria and Acidobacteria decreased significantly (Fig. 3B).

The PCoA revealed that the microbial community varied significantly with soil depth (Appendices Table A2) at both the phylogenetic (Fig. 3C, weighted Unifrac, ANOSIM R = 0.566, *P* = 0.001; ADONIS R^2^ = 0.328, *P* = 0.001) and species level (Fig. 3D, Bray-Curtis ANOSIM R = 0.759, *P* = 0.001; ADONIS R^2^ = 0.500, *P* = 0.001). In addition, the variation of the microbial community in response to all the disturbances significantly differed from that of the *in-situ* soil for layer I (ANOSIM R = 0.315, *P* = 0.022; ADONIS R^2^ = 0.152, *P* = 0.024), whereas such difference between disturbed and *in-situ* soils was not detected in the deeper layers (Appendices Table A3).

**Figure 4:**
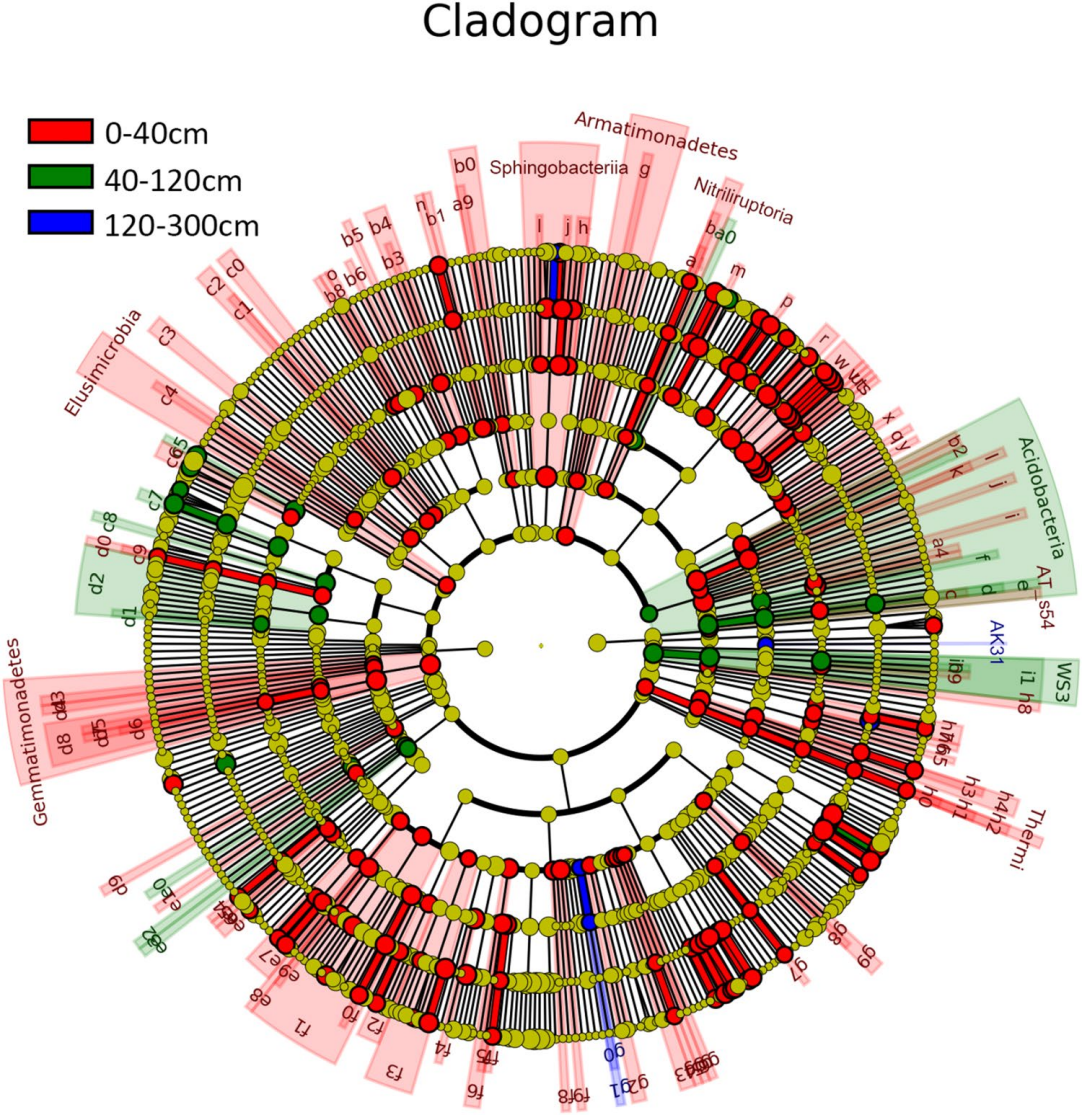
Least discriminant analysis (LDA) effect size taxonomic cladogram comparing all the disturbed soils and *in-situ* soil for three depth layers. Significantly discriminant taxon nodes are colored (red indicating layer I, green layer II, blue layer III). The yellow nodes indicate non-significant taxa among the three layers. Each circle’s diameter is proportional to the taxon’s abundance. Highly abundant and selected taxa are indicated: (**a**) Nitrospiraceae; (**b**) Nitriliruptorales; (**c**) Koribacteraceae; (**d**) Acidobacteriales; (**e**) Acidobacteria; (**f**) Acidobacteria; (**g**) Fimbriimonadetes; (**h**) Chitinophagaceae; **(i**) Chloracidobacteria; (**j**) Flammeovirgaceae; (**k**) Sva0725; (**l**) Rhodobacteraceae; (**m**) Streptosporangiaceae; (**n**) Caldilineales; (**o**) Chloroflexaceae; (**p**) Promicromonosporaceae; (**q**) lamiaceae; (**r**) Microbacteriaceae; (**s**) Geodermatophilaceae; (**t**) Glycomycetaceae; (**u**) Intrasporangiaceae; (**v**) Kineosporiaceae; w. Microbacteriaceae. For the complete list of discriminate taxa and ranks used to generate this cladogram, please refer to Dataset S1.

**Figure 5:**
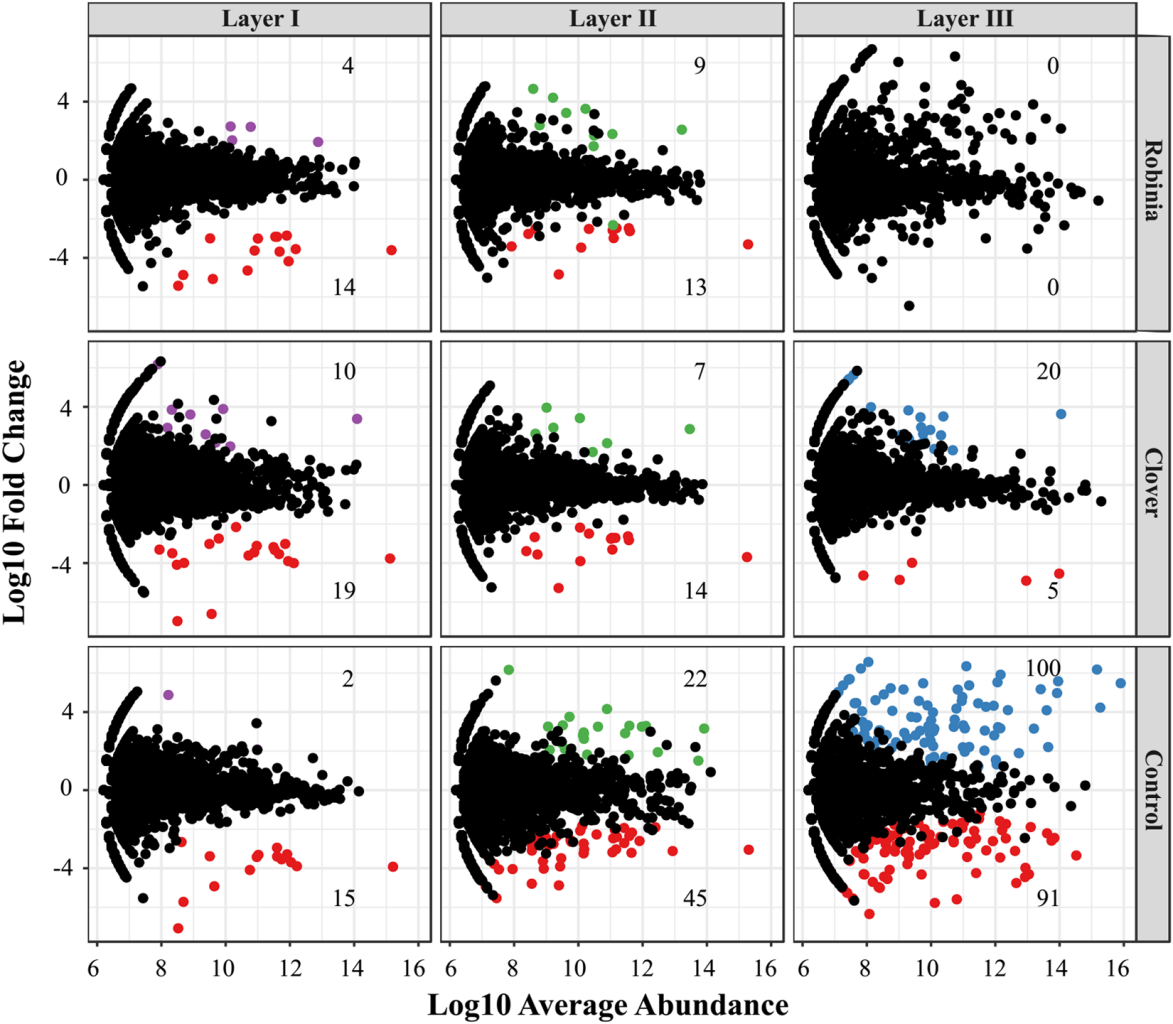
Enrichment and depletion of the 4826 OTUs included in the greenhouse experiment for each disturbance treatment group per soil layer as compared with the *in-situ* soil. The points in red color represented the depleted OTUs. The points in purple, green and blue color represented the enriched OTUs in layer I, layer II and layer III, respectively. For the complete list of discriminate taxa, please refer to Dataset S1.

Significant taxonomic differences (biomarks) among the layers were examined with Lefse (Fig. 4, Appendices Dataset A1). Different taxonomic representatives of statistically and biologically consistent OTUs were different among the three layers. The detailed descriptions of significant taxa among the layers were in the Appendices Result 1. To identify the distinct OTUs in each treatment, we conducted a differential OTU abundance analysis. By using the OTU counts from the *in-situ* soil as a contrast and an adjusted *P* value cutoff of 0.05, we found that the number of differentially abundant OTUs were similar between the Robinia and Clover treatments in layers I and II (Appendices Fig. A2, Fig. 5); however, differential OTUs were not detected in the Robinia treatment in layer III. Meanwhile, there were 100 OTUs enriched, and 91 OTUs depleted in the Control (unplanted) treatment in layer III (Fig. 5). The detailed descriptions of significant taxa among the layers were in the Appendices Result 2.

**Figure 6:**
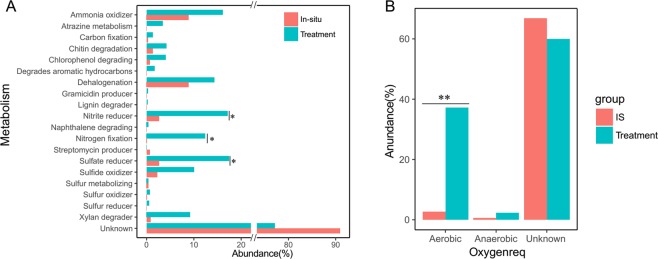
The metabolic functions (**A**) and oxygen requirements (**B**) of the identified genera in the disturbance treatments (Robinia, Clover, and Control) and *in-situ* soil, as analyzed by the METAGENassist database. ANOVA test: *P < 0.05, **P < 0.01.

The metabolic functions and oxygen requirements of the identified genera were predicted through the METAGENassist database tool. Most metabolic functions in the disturbed treatments exceeded those of *in-situ* soil (Fig. 6A). Nitrite reduction (17.1%), nitrogen fixation (12.4%), and sulfate reduction (17.4%) were significantly greater in disturbed soils when compared with their *in-situ* values (2.6%, 0.09%, and 2.6%, respectively). There were more aerobic microbial genera (Fig. 6B) in the disturbed soils (37.2%) than in the *in-situ* soil (9.4%, *P* < 0.01).

### Role of soil properties in shaping microbial community

Our study showed that pH, SOM, TN, AN, AK, and AP varied significantly down through the soil profiles (Table 1 and Appendices Fig. A4). SOM, TN, AK, and AP, decreased with depth (Table 1), whereas pH tended to increase with depth, going from pH 8.11 to 8.33 in disturbed soils, and likewise from 8.36 to 8.71 in the *in-situ* soil.

**Table 1:** Physical and chemical properties of the disturbed soils and *in-situ* soils at three depth layers.

	Layer	pH	SOM (g/kg)^†^	TN (g/kg)	AN (mg/kg)	AK (mg/kg)	AP (mg/kg)
Disturbed soils	I	8.11 ± 0.10a	11.87 ± 1.80a	0.50 ± 0.08a	46.76 ± 17.99a	108.68 ± 10.41a	4.88 ± 2.16a
	II	8.25 ± 0.10b	8.69 ± 0.51b	0.38 ± 0.05b	29.97 ± 10.70b	125.16 ± 8.26b	2.40 ± 0.73b
	III	8.33 ± 0.08c	6.97 ± 1.40c	0.30 ± 0.09c	25.81 ± 13.86b	91.21 ± 20.07c	2.54 ± 0.87b
*In situ* soils	I	8.36 ± 0.24a	10.94 ± 1.93a	0.55 ± 0.05a	26.69 ± 11.48a	102.68 ± 14.94a	7.55 ± 2.84a
	II	8.67 ± 0.13b	7.78 ± 0.64b	0.44 ± 0.01b	15.31 ± 3.85b	101.93 ± 5.50a	3.29 ± 0.37b
	III	8.71 ± 0.15b	6.45 ± 1.09c	0.27 ± 0.05c	10.50 ± 2.51c	71.83 ± 14.60b	2.58 ± 0.58b

Layer I: 0–40 cm; II: 40–120 cm; III: 120–300 cm.

^†^SOM: soil organic matter; TN: total nitrogen; AN: available nitrogen; AP: available phosphorus; AK: available potassium.

^abc^Values for each physicochemical variable that do not share the same letter within disturbed or *in-situ* soils are significantly different (at *P* < 0.05).

**Figure 7:**
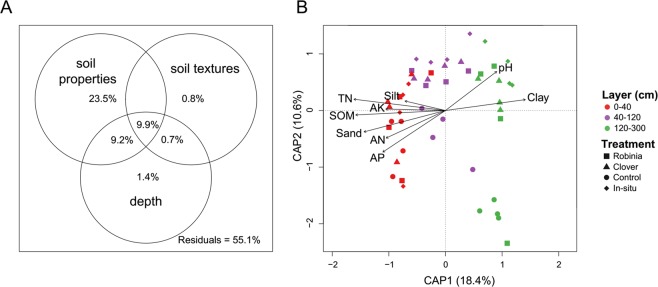
(**A**) Variation partitioning analysis of the microbial communities by soil properties, texture, and depth. (**B**) Constrained analysis of principal coordinates (CAP) based on the Bray-Curtis matrix. SOM: soil organic matter; TN: total nitrogen; AN: available nitrogen; AP: available phosphorus; AK: available potassium. Treatments: RD: Robinia; CD: Clover; WD: Control; IS:*in-situ*.

To quantify the contribution of soil chemical properties, texture, and depth to the microbial community structure, we performed a variation partitioning analysis (Fig. 7A). Together, all variables (soil chemical properties, texture, and depth) could explain 44.9% of the variation in the microbial communities. Soil properties alone explained the most variation (23.5%) found in the microbial communities. The simultaneous contribution of all variables across to all the three layers was only 9.9% (Fig. 7A). The CAP, based on Bray-Curtis metrics, suggested that the soil properties were significantly related to the microbial community (*P* < 0.001, Fig. 7B), and a similar amount of variation was explained when using the weighted UniFrac (*P* < 0.001, Appendices Fig. A3). This demonstrated that pH (ADONIS R^2^ = 0.084, *P* = 0.003), SOM (ADONIS R^2^ = 0.087, *P* = 0.003), AP (ADONIS R^2^ = 0.133, *P* = 0.001), and AK (ADONIS R^2^ = 0.046, *P* = 0.031) were the main environmental variables correlated with the structure of microbial community; by contrast, soil texture had little influence. Meanwhile, the PERMANOVA analysis for each layer showed that AK played an important role in shaping microbial community in layer I, while pH, TN, AK, and AP significantly affected the microbial composition in the layer II, without any effects detected for layer III (Appendices Table A4).

Specifically, the relative abundance of Proteobacteria was negatively correlated with pH (Spearman *rho* = –0.483, *P* < 0.05) (Appendices Table A5), whereas this association was not significant in other dominant lineages, such as Actinobacteria and Acidobacteria (respectively, Spearman *rho* = 0.269, –0.453). The Acidobacteria, Chloroflexi, and Planctomycetes were positively correlated with SOM (*P* < 0.05) and TN (*P* < 0.05; Appendices Table A5). Notably, while the relative abundance of the candidate phylum GAL15 increased with the soil depth, it was significantly negatively correlated with SOM, TN, AK, and AP. This result suggested that GAL15 was sensitive to variation in most of the soil properties.

## Discussion

### Microbial diversity in different soil depths related to soil properties

We found that microbial α-diversity decreased with soil depth. In particular, a higher richness and diversity were observed in the disturbed than *in-situ* soil. This is consistent with previous studies finding that microbial α-diversity decreased from native tropical forest to soils disturbed by deforestation and soybean cultivation^44^. Hierarchical clustering based on the weighted UniFrac distance indicated that the major factor influencing the variation in the microbial community was soil depth (Fig. 1B). Steven, *et al*.^12^ found that soil microbial communities were highly stratified vertically in topsoil (0–5 cm) of dryland yet showed less variation at differing horizontal spatial scales.

Soil chemical properties, nutrient availability, and texture are known to vary considerably through soil profiles spanning the ground surface, vadose zone, and saturated soil^45^. In our study, soil properties were the main driver of microbial community assembly, while the respective contribution of the soil properties depended on soil depth. More nutrients would be expected to facilitate the growth of particular microorganisms^46^. Prior work has demonstrated that the microbial community is sensitive to nitrogen and phosphorous inputs, leading to increased relative abundances of the faster-growing, copiotrophic bacterial taxa^47^. Indeed, we observed that nutrient factors (e.g., TN, AK, and AP) significantly affected the microbial compositions in upper soil layers (0–120 cm depth). However, no soil properties were detected to influence the structure of microbial communities in lower soil layers (120–300 cm depth). This could be explained that soil nutrients, including SOM, TN, and AP, had vertical distributions in soil, with almost all nutrients concentrated most in the topsoil across the soil profiles^8,18^.

Our results demonstrated that the relative abundance of Actinobacteria increased significantly within the soil profiles in the disturbance treatments (especially Robinia), but it did not exhibit consistent shifts in the *in-situ* soil. Other work reported that the abundance of Actinomycetes was lowest in the top 5 cm of soil but increased significantly at a 2-m depth in terrace soil^9^. Nevertheless, contrary results for profile changes in Actinobacteria were found in grassland soils^13^, with long-term fertilization^22^, and in a forested montane watershed^10^. Proteobacteria and Acidobacteria significantly decreased through the soil profiles, though they were ubiquitous and abundant members across all the samples. The phylum Proteobacteria is known to prefer eutrophic and facultative anaerobic environments^24,48^, which may partially explain our results. Additionally, Acidobacteria was negatively correlated with pH, which increased with soil depth; this was supported by a few previous studies^13,49,50^.

### Important microbes in different layers are linked to soil ecosystem functioning

Many microbial taxa likely play key roles in assembling microbial communities and driving nutrient cycling^10^. In the present study, distinct microbes were distinguished to occur in the different soil layers by the Lefse analysis. A recent study showed that not only the predominant microbes but also several deeper taxa with low abundances might participate significantly in ecosystem functioning, such as Nitrospira, Planctomycetes, Firmicutes, and Verrucomicrobia^51^. Here, we found that the *Nitrospira* abundance was higher in the subsoil than topsoil, and positively correlated with AP. Nitrification is a key process in the biogeochemical nitrogen cycle, in which Nitrospira are the crucial functional bacteria in tillage and no-tillage land systems, and the predominant nitrite-oxidizing bacteria in paddy soils^52–54^. Further work has since shown that *Nitrospira* species (termed ‘comammox’) are capable of complete nitrification through the oxidation of ammonia via nitrite to nitrate by a single microorganism, rather than by two distinct microorganisms^55,56^.

Planctomycetes, an anaerobic ammonium-oxidizing bacterium, is also related to the nitrogen cycle^52^, and showed a decreased relative abundance with soil depth in our study. The Firmicutes taxon was heavily depleted by plant disturbance when compared with its *in-situ* soil abundance. Other research has suggested that Firmicutes can exist in nutrient-poor environments^22^, which may explain the depletion of Firmicutes in the greenhouse experiment under higher nutrient conditions (i.e., of SOM, AN and AK) than found *in-situ* soil. Furthermore, we found that Verrucomicrobia occurred in two soil layers (0–40 cm and 120–300 cm). Several studies reported that Verrucomicrobia are ubiquitous and present in low relative abundance along soil nutritional gradients^57–59^, yet reaching their peak abundance in soil 25–50 cm deep^15^. Compared with that in the *in-situ* soil, the abundance of Verrucomicrobia was higher under the plant-disturbed soil; this may be explained by reductions in TN and increases in SOM^60,61^.

### Impacts of disturbances on the metabolism of microbial communities

Microbes are an important component of soil, and play key roles in nutrient turnover and fixation, including those of carbon, nitrogen, and phosphorous. The prediction of diverse metabolic functions and oxygen requirements of the identified genera gave us a functional snapshot of the microbial community^62^. Our results showed that most metabolic activities were higher in disturbance treatments than in the *in-situ* soil. Among them, the abundance of nitrogen-fixing groups was significantly increased; this may be related to the legume growth by Robinia and Clover, species capable of forming nitrogen-fixation nodules with rhizobium. Similarly, the nitrite reducers were also increased, which could convert nitrite to a gaseous product (either NO, N_2_O, or N_2_) and thus play a crucial role in denitrification. Finally, as both sulfate reducers and sulfide oxidizers significantly increased when compared with the *in-situ* soil, this result suggests that sulfur cycling was likely affected by the soil disturbance treatments.

Our results also showed that disturbance accumulated the potential microbial functions related to pesticides metabolism, for example atrazine metabolism and degrades aromatic hydrocarbons (Fig. 6A). This may be related to the predicted higher number of aerobic bacteria and higher metabolic activities in the disturbed soils. For example, the slightly increased abundance of chitin, xylan, and lignin degraders, and even those metabolizing atrazine, chlorophenol, and dehalogenation, corresponded to the increased abundance of aerobic genera under disturbance. Our soil samples were collected in late July from a wheat-corn cropping rotation, where many pesticides and herbicides were applied annually in May and June; an increasing atrazine metabolism could be explained by an increasing aerobic microbial mineralization^63^. Given that a large proportion of unknown metabolic functions were not changed, this suggests the microbial community contribution towards maintaining the soil ecosystem functions was not sensitive to disturbances. A potential limitation in the interpretation of our findings should be considered: our results were based on the data come from pot experiment and could not represent that occur *in situ* at deep soils.

## Conclusions

Our study showed significant shifts occurring in the microbial community through a 3-m soil depth profile from cultivated land samples treated with legume growth and air exposure disturbances. Some significant species, such as those belonging to Nitrospira, Verrucomicrobia, and Planctomycetes, may be of great importance for supporting the structure and function of the soil microbial community. The abundance of potentially aerobic microbial genera was improved greatly by the disturbances. These findings reveal the complex dynamics of soil microbial communities and nutrient properties to abiotic and biotic disturbances, which helps predict how microbiomes through soil profiles are likely to respond to current and future environmental changes in agricultural ecosystems, especially for the arable soil degradation via deep tillage, water and soil erosion.

## Supplementary information

Supplementary material

Dataset 1

Dataset 2
