# Supplementary material for: Subsoil microbial community responses to air exposure and legume growth depend on soil properties across different depths
Source: Sci Rep. 2019 Dec 6;9:18536. doi: 10.1038/s41598-019-55089-8 (PMC6898284; doi:10.1038/s41598-019-55089-8)

**Supplementary materials**

**Subsoil microbial community responses to air exposure and legume growth depend on soil properties across different depths**

**Fan Yang • Ziheng Peng • Jiamin Gao • Hongmei Yan • Weimin Chen***

*State Key Laboratory of Crop Stress Biology in Arid Areas, College of Life Sciences, Northwest A&F University, Yangling, Shaanxi 712100, P. R. China*

***Supplementary Result1***

In layer I, the abundant groups were the classes Chloracidobacteria, Sphingobacteriia, and Gemmatimonadetes, the families Erythrobacteraceae, Geodermatophilaceae, Microbacteriaceae, Micromonosporaceae, Intrasporangiaceae, Gemmatimonadetes, and Flammeovirgaceae, and the genera *Skermanella*, *Steroidobacter*, *Catellatospora*, *Agromyces*, and *Pontibacter*. In particular, the nitrogen-fixing Bradyrhizobiaceae was significantly abundant in layer I.

In layer II, the significantly different occurring taxa included the phyla Firmicutes, Planctomycetes, Nitrospirae, the orders Exiguobacterales, Clostridiales and Acidobacteriales, and the families Nitrosomonadaceae, Alicyclobacillaceae, Veillonellaceae, and Planococcaceae. In layer III, the abundant taxa were Verrucomicrobia and the archaea Crenarchaeota at the phyla level, as well as the family Nitrosomonadales.

***Supplementary Result2***

The OTUs belonging to the orders Bacillales and Exiguobacterales in the phylum Firmicutes were depleted by every treatment in layer I (Appendices **Dataset A2**). However, several members within the Alpha-, Beta-, and Gamm-proteobacteria were enriched in each layer (Appendices **Dataset A2** and **Fig. A2**). For example, the OTUs belonging to the classes Acidimicrobiia and Thermoleophilia were mainly enriched by the Control (water), while the Acidobacteria-6 and RB25 classes were depleted in the soils below 40 cm. Members belonging to the genera *Nitrospira* and *JG37*-*AG*-*70* in the Nitrospiraceae were depleted in deeper soil (i.e., 120–300 cm) (**Dataset A2**, **Fig. 5**).

**Table A1** Soil texture of the *in-situ* soils with different depths, determined via particle size distribution, including the percent composition of clay, silt, and sand.

| **Depth (cm)** | **Sand (%)** | **Silt (%)** | **Clay (%)** |
| --- | --- | --- | --- |
| 0-5 | 8.62 | 72.19 | 18.84 |
| 5-10 | 10.90 | 69.66 | 19.44 |
| 10-20 | 9.94 | 70.18 | 19.88 |
| 20-40 | 6.62 | 71.91 | 21.47 |
| 40-60 | 3.78 | 68.30 | 27.92 |
| 60-80 | 3.42 | 70.08 | 26.51 |
| 80-100 | 4.18 | 72.42 | 23.40 |
| 100-120 | 3.22 | 69.92 | 26.86 |
| 120-150 | 2.65 | 69.56 | 27.79 |
| 150-200 | 3.08 | 68.62 | 28.30 |
| 200-250 | 2.69 | 68.40 | 28.91 |
| 250-300 | 3.05 | 69.51 | 27.44 |

**Table A2** Permutational MANOVA and ANOSIM analyses of the microbial diversity among different soil depth layers. Layer I: 0–40 cm; II: 40–120 cm; III: 120–300 cm

| **Layer** | **ADONIS** | |  | **ANOSIM** | |
| --- | --- | --- | --- | --- | --- |
|  | **R^2^** | ***P*** |  | **R** | ***P*** |
| I vs II | 0.236 | 0.001 |  | 0.570 | 0.001 |
| I vs III | 0.324 | 0.001 |  | 0.678 | 0.001 |
| II vs III | 0.228 | 0.001 |  | 0.470 | 0.001 |

**Table A3** Permutational MANOVA and ANOSIM analyses of the microbial diversity between the disturbed soils and in-situ soils at three depth layers. Layer I: 0–40 cm; II: 40–120 cm; III: 120–300 cm

| **Layer** | **ADONIS** | |  | **ANOSIM** | |
| --- | --- | --- | --- | --- | --- |
|  | **R^2^** | ***P*** |  | **R** | ***P*** |
| Disturbed I vs in-situ I | 0.152 | 0.024 |  | 0.315 | 0.022 |
| Disturbed II vs in-situ II | 0.129 | 0.053 |  | 0.175 | 0.132 |
| Disturbed III vs in-situ III | 0.153 | 0.071 |  | 0.078 | 0.223 |

**Table A4** Effects of the predominant soil properties on the microbial diversity in soil layers, based on the Bray-Curtis matrix used in a permutational MANOVA analysis (ADONIS). Layer I: 0–40 cm; II: 40–120 cm; III: 120–300 cm.

|  | **Layer I** | |  | **Layer II** | |  | **Layer III** | |
| --- | --- | --- | --- | --- | --- | --- | --- | --- |
|  | **R^2^** | ***P*** |  | **R^2^** | ***P*** |  | **R^2^** | ***P*** |
| pH | 0.098 | 0.078 |  | 0.116 | 0.020* |  | 0.137 | 0.091 |
| SOM† | 0.048 | 0.337 |  | 0.074 | 0.122 |  | 0.137 | 0.077 |
| TN | 0.074 | 0.142 |  | 0.120 | 0.014* |  | 0.039 | 0.639 |
| AN | 0.038 | 0.578 |  | 0.053 | 0.318 |  | 0.102 | 0.175 |
| AK | 0.257 | 0.002** |  | 0.159 | 0.002** |  | 0.032 | 0.765 |
| AP | 0.096 | 0.062 |  | 0.105 | 0.023* |  | 0.038 | 0.663 |
| Clay | 0.026 | 0.848 |  | 0.033 | 0.598 |  | 0.014 | 0.994 |
| Silt | 0.042 | 0.506 |  | 0.047 | 0.381 |  | 0.055 | 0.459 |
| Sand | 0.032 | 0.712 |  | 0.034 | 0.610 |  | 0.073 | 0.314 |

† SOM: soil organic matter; TN: total nitrogen; AN: available nitrogen; AP: available phosphorus; AK: available potassium

* *P* < 0.05; ** *P* < 0.01

**Table A5** Spearman’s correlation coefficients (*rho* values) between microbial phyla and the six soil physical and chemical properties

| **Phylum** | **pH** | **SOM**† | **TN** | **AN** | **AK** | **AP** |
| --- | --- | --- | --- | --- | --- | --- |
| Actinobacteria | 0.269 | –0.348 | –0.405 | –0.151 | 0.108 | 0.021 |
| Proteobacteria | –0.483* | 0.314 | 0.161 | 0.393 | –0.092 | –0.042 |
| Acidobacteria | –0.453 | 0.653** | 0.636** | 0.391 | 0.200 | 0.160 |
| Firmicutes | 0.316 | –0.007 | 0.200 | –0.355 | 0.194 | 0.250 |
| GAL15 | 0.406 | –0.64** | –0.608** | –0.447 | –0.564** | –0.557** |
| Nitrospirae | 0.303 | –0.232 | –0.220 | –0.282 | –0.039 | –0.583** |
| Chloroflexi | –0.140 | 0.459* | 0.509* | 0.225 | 0.547** | 0.234 |
| Gemmatimonadetes | –0.002 | 0.190 | 0.254 | –0.038 | 0.098 | 0.003 |
| Planctomycetes | –0.476* | 0.622** | 0.571** | 0.493* | 0.399 | 0.082 |
| Cyanobacteria | –0.201 | 0.334 | 0.240 | 0.149 | 0.182 | 0.073 |

† SOM: soil organic matter; TN: total nitrogen; AN: available nitrogen; AP: available phosphorus; AK: available potassium

* *P* < 0.05; ** *P* < 0.01

**Fig. A1** Bacterial relative abundance and local occupancy were positively correlated (Spearman, *rho* = 0.9850, *P* < 0.01). The abundances were transformed into presence-absence (1-0) data, containing 4770 OTUs


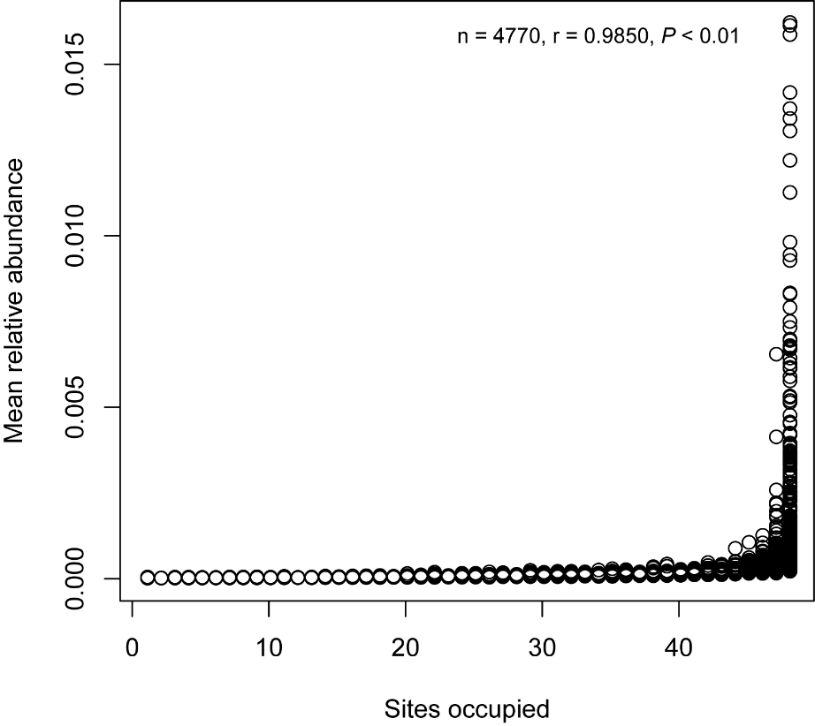


**Fig. A2** Differentially abundant operational taxonomic units (OTUs) in the three layers of disturbed (robinia and clover) soils and in-situ (control) soils

**
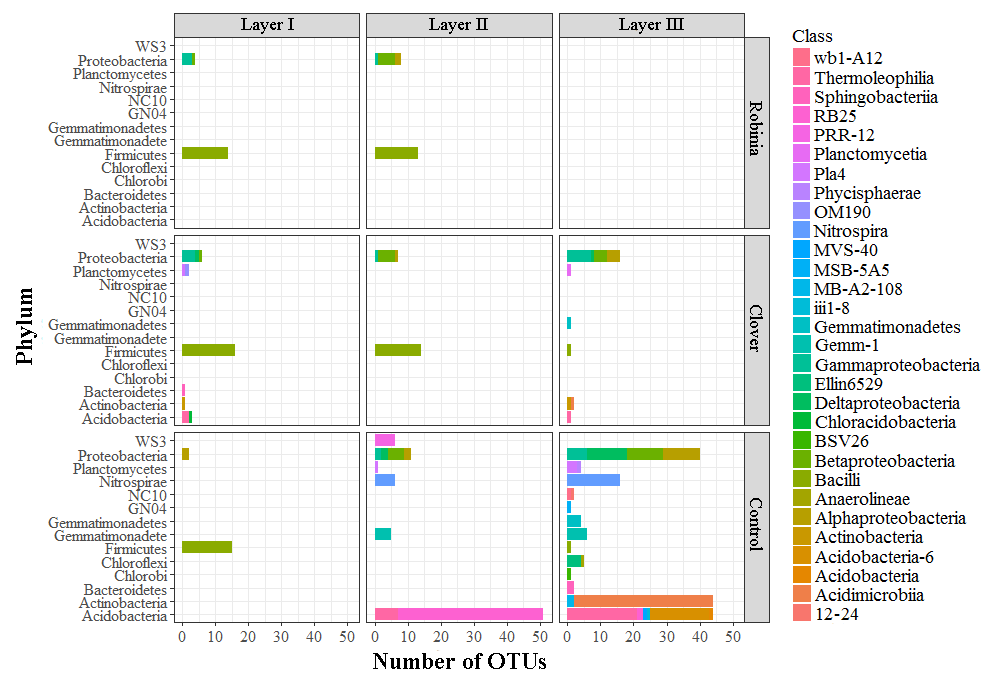
**

**Fig. A3** Constrained analysis of principal coordinates (CAP) based on the weighted UniFrac confirmed that the variation in microbial community with depth was explained by soil properties.


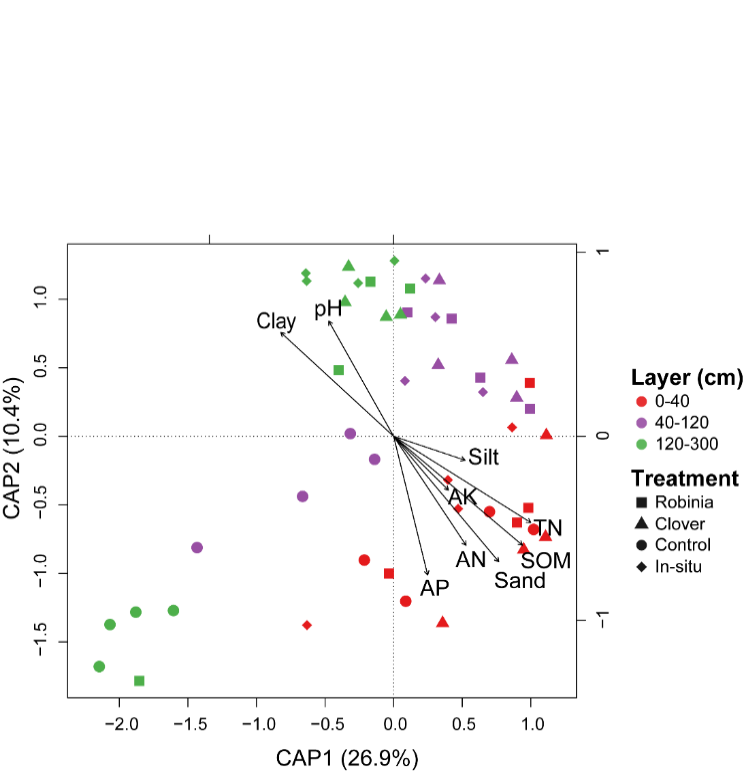


**Fig. A4** Pearson *r* linear correlations among the soil properties. Blue indicates a positive correlation, whereas red indicates a negative correlation.


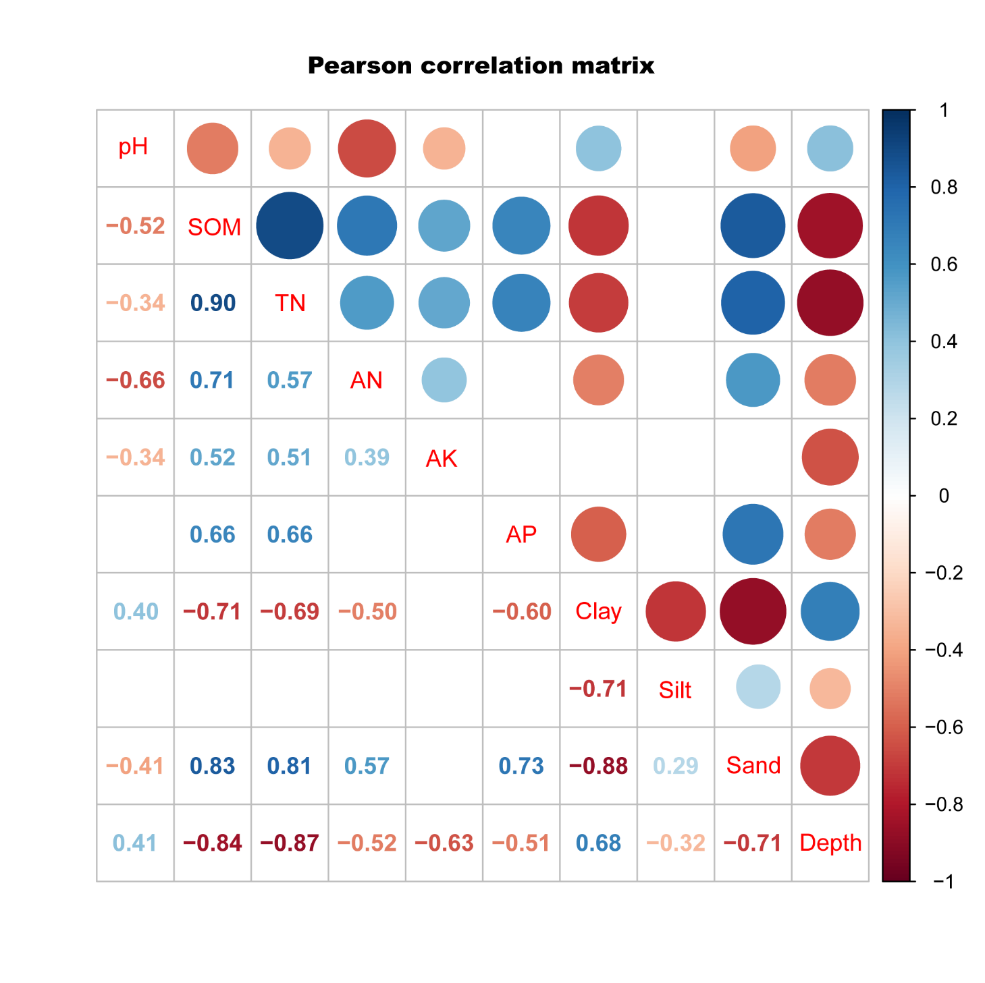


**Fig. A5** Oxygen requirements of the identified genera between the disturbed soils (Robinia, Clover, and Control) and the in-situ soil in three layers, as analyzed by the METAGENassist database. ANOVA test: * *P* < 0.05, ** *P* < 0.01


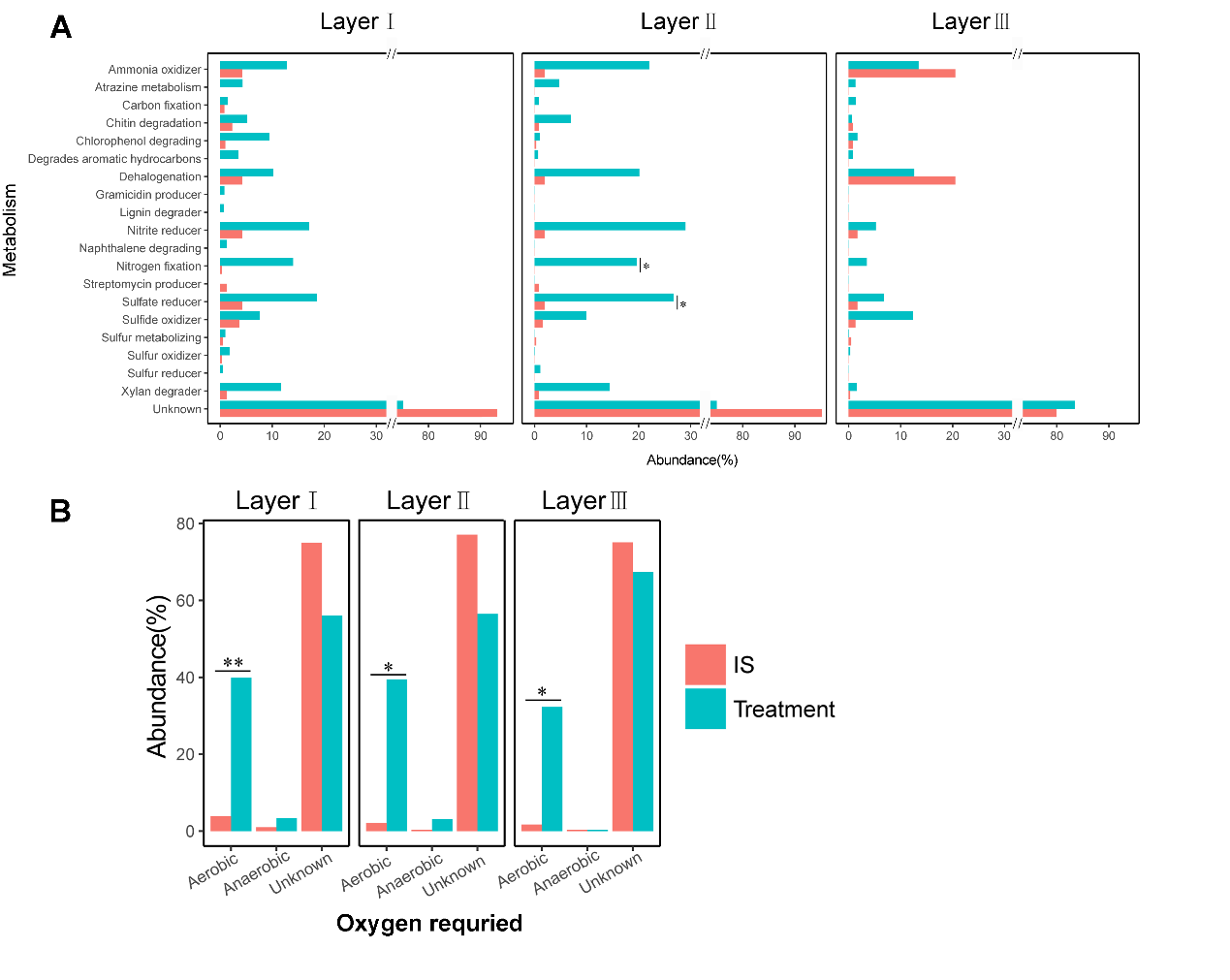

Supplement: Supplementary file 1 — Supplementary material [file 41598_2019_55089_MOESM1_ESM.docx]
